# Genomic resources and genetic improvement of vital tropical and subtropical fruit crops: current status and prospects

**DOI:** 10.1093/aobpla/plaf059

**Published:** 2025-10-17

**Authors:** Anupama Roy, Tilak Chandra, Raju Mondal, Johiruddin Molla, Sarika Jaiswal, Manish Srivastava, Dinesh Kumar, Kutubuddin A Molla, Mir Asif Iquebal

**Affiliations:** Division of Agricultural Bioinformatics, ICAR-Indian Agricultural Statistics Research Institute, New Delhi 110012, India; The Graduate School, ICAR-Indian Agricultural Research Institute, New Delhi 110012, India; Division of Agricultural Bioinformatics, ICAR-Indian Agricultural Statistics Research Institute, New Delhi 110012, India; Central Sericultural Germplasm Resources Center, Central Silk Board-Ministry of Textiles (GoI), Hosur, Tamil Nadu 635109, India; Department of Botany, Ghatal Rabindra Satabarsiki Mahavidyalaya, Ghatal, Paschim Medinipur, West Bengal 721212, India; Division of Agricultural Bioinformatics, ICAR-Indian Agricultural Statistics Research Institute, New Delhi 110012, India; Division of Fruits and Horticultural Technology (FHT), ICAR-Indian Agricultural Research Institute, New Delhi 110012, India; Division of Agricultural Bioinformatics, ICAR-Indian Agricultural Statistics Research Institute, New Delhi 110012, India; Crop Improvement Division, ICAR-Central Rice Research Institute, Cuttack, Odisha 753006, India; Division of Agricultural Bioinformatics, ICAR-Indian Agricultural Statistics Research Institute, New Delhi 110012, India; Molecular Function & Environment

**Keywords:** tropical and subtropical fruits, genomic resources, quantitative trait loci, functional genomics, miRNAs, gene editing, multi-omics web resources

## Abstract

Fruits are integral to agriculture and receive considerable attention due to their multifold health and nutritional benefits, particularly in the post-pandemic era. The wide range of climatic conditions gives rise to a myriad of fruits grown in different agro-climatic zones; however, fruits grown in tropical and subtropical zones deserve particular attention by virtue of their bountiful nutritional compounds and contribution to substantial growth in the economic sector. Nevertheless, their production is severely affected by their perishable and delicate nature, often limited by various biotic and abiotic factors that result in pre- and post-harvest losses. Scientific advancements have catalyzed efforts to augment the production of tropical and subtropical fruits through genetic and genomic interventions, resulting in the development of numerous advanced genomic resources. These innovations present new opportunities to address key challenges in fruit production, including the mitigation of anti-nutritional factors, improvement of sensory attributes, extension of both pre- and post-harvest shelf-life, chilling sensitivities, and ancillary crop improvements. This review provides a comprehensive synthesis of the genetic and genomic resources available for influential tropical and subtropical fruits, with an emphasis on their potential impact in the context of market acceptability and economic feasibility. These include whole-genome sequencing, which provides insights into domestication and adaptation processes; quantitative traits facilitating the identification of loci associated with desirable traits; functional genomics, enabling biotechnological interventions; the miRNA repertoire for precise trait modulation; and the integration of these resources with CRISPR/Cas9 for tailoring trait modification and recovery. Furthermore, the review highlights the role of web-based platforms that enhance stakeholder engagement and marketing strategies, thereby accelerating the translational potential of research and development in this field. Moreover, the inclusion of single-cell approaches for uncovering cellular heterogeneity, along with multi-omics strategies for dissecting complex traits, is critically discussed. Collectively, these genomic resources are poised to drive transformative changes in the production and utilization of tropical and subtropical fruits, contributing to global nutritional security and sustainable horticultural practices.

## Teaser

Tropical and subtropical fruits are nutrient-rich and essential for human health, yet their low production limits their availability for growing populations. The pandemic underscored their importance as diets shifted toward healthier choices. Advances in genetic and genomic research offer auxiliary wealth for augmenting their production and new opportunities to boost fruit yield, enhance flavour, aroma, nutritional value and stress management. By harnessing these resources, scientist can develop improved fruit varieties that support sustainable health and nutrition, even under environmental stresses. This approach holds promise for securing food systems and promoting well-being in the face of global challenges.

## Graphical Abstract

**Graphical Abstract:**
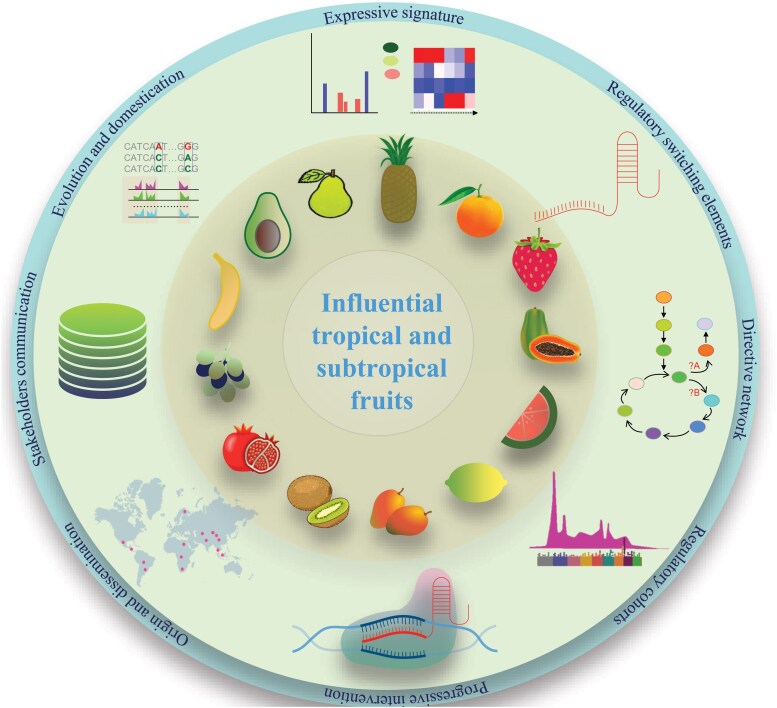

## Introduction

Horticultural products have significantly contributed to recent advances in food, nutrition, health, and socioeconomic development (Singh et al. 2022). Fruits are amongst the choicest of nature’s gifts to humankind and account for a substantial fraction of the world's agricultural output in terms of nutritional values and per capita availability (Krishnaswamy and Gayathri 2018, Singh et al. 2022). Besides being edible and nutritious, fruits have also acquired extensive symbolic and cultural significance (Savo et al. 2016). Fruit crops are often classified based on climate adaptability, diverse agroecological zones, and other characteristics such as tree growth habit, inflorescence type, cotyledon structure, morphology and physiology, respiratory behaviour, photoperiodic responses, tolerance to biotic and abiotic stresses, longevity, consumer preference, bioactive compound content, and genomic relationships. Among these, climate and agroecological criteria generally predominate in classification (Pennington and Fisher 2009, Krishnaswamy and Gayathri 2018). Therefore, there are two kinds of fruits broadly classified based on ambient temperature requirements, climatic adaptation, growth, and sustainability, namely, tropical and temperate (Biale 1961, Mitra 2014). Subtropical fruits are a third type of fruit crop grown under climatic conditions ranging between temperate and tropical (Atkinson 2011). Both tropical and subtropical fruits are characterized by their specific ecological requirements, making them rich sources of essential nutrients, vitamins, minerals, antioxidants, and other bioactive compounds. These attributes confer multiple health benefits, including enhanced immune function, reduced oxidative stress, and improved cardiovascular health (Mohamed et al. 2011). These are integral to global trade, contributing significantly to the economies of their producing countries while supporting diverse agricultural practices that promote environmental sustainability (Kader and Yahia 2011). Prime commercially important tropical and subtropical fruits, including avocado, banana, citrus, grapes, guava, kiwifruit, lychee, mango, oranges, papaya, pomegranate, pineapple, strawberry, and watermelon, are climate-specific; some of the fruits may be grown in more than one climate. For example, mango is principally a tropical fruit crop that thrives well in both tropical and subtropical climates (Kader and Yahia 2011, Mohamed et al. 2011). Grapes and strawberries can be grown in both temperate and subtropical regions (Biale 1961, Atkinson 2011, Lustosa da Silva et al. 2023). As they are grown in diverse habitats, they offer unique flavours and nutritional content; however higher levels of production are needed to support the demand of the burgeoning population, but their distinct mechanisms of adaptation, specific requirements for growth, diversified developmental patterns, non-adaptation to extreme climate, short pre- and post-harvest life, and several species-specific ancillary bottlenecks limits their post-harvest utilization and consequently reduce trade values in international commerce. Moreover, owing to their vast genetic and species diversity, these fruits often fail to display their full potential productivity, being constrained by various environmental cues. For instance, chilling sensitivity represents a major physiological limitation in many of these fruits, severely impairing cold-chain storage, post-harvest longevity, and international marketability (Roussos 2024).

**Figure 1.**
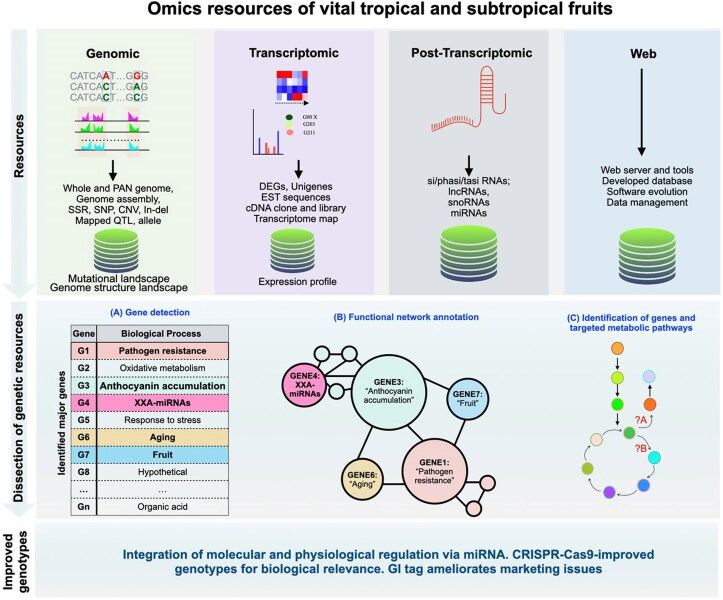
Summarized particulars to delineate the processes involved in enhancing sustainability in tropical and subtropical fruit crop improvement through the utilization of various biological resources, including genomics, transcriptomics, post-transcriptomics, web-based, and functional gene network analysis. Genomics enables comprehensive analysis of whole and PAN genome-assisted assembly, as well as the identification of genetic variations like single nucleotide polymorphism (SNP), SSR, CNV, and In-del in genomic landscapes. Transcriptome landscapes provide unigenes, DEGs, EST sequences, and many more signature repertoires. Post-transcriptomics dissects mostly regulatory switches, uncovering mi/si/phasi/ta-si RNA. Web resources are prerequisites for web server and database development and management for easy handling of large omics data. All-inclusive integrated resources empower gene/trait discovery, functional expression, co-expression network analysis, and identification of targeted metabolic pathways to enhance specific traits in fruit genotypes.

Scientific interventions are imperative to augment breeding initiatives and overcome existing bottlenecks, such as intertwined climatic, ecological, economic, and technological factors, thereby expanding the desired breeding objectives on a local and global scale (Irfan et al. 2023, Sarkar et al. 2023). To ensure the fastest growth in the fruit and horticulture sectors, the integration of advanced genetic and genomic resources is crucial for developing fruit varietal wealth, hi-tech production techniques, and converting translational processed commodities, which should be imperative to accelerate trait interventions and monetary benefits. Nevertheless, unlike major staple crops, fruit crops have lagged behind in breeding and biotechnological advancements due to a lack of precise genetic tools, technologies, and underlying assisted genetic and genomic resources (Mathiazhagan et al. 2021, Irfan et al. 2023). In the recent era, the revolution in genomics and its utilization in the improvement of fruit crops have opened new opportunities for solving the problems encountered in fruit crop cultivation and ensuring nutritional security for the consuming population, thereby developing a wealth of genomic resources and their assisted genetic improvement strategy for tropical and subtropical fruit cultivation, popularizing their consumption, and addressing nutritional security concerns (Mathiazhagan et al. 2021, Irfan et al. 2023, Sarkar et al. 2023). This review advocates for a comprehensive approach to understanding prime tropical and subtropical fruits, their origin and distribution, and how to bolster their cultivation through the revitalization of genomic resources, encompassing whole-genome sequencing for expedited evolution and domestication events, quantitative trait loci (QTL) mapping for the identification of genetic variants for complex traits, signature transcript analysis for delineating gene expression patterns and regulatory networks, miRNA-mediated regulation for complexity and precision in gene tuning, transformative CRISPR/Cas9 for precise editing, and multi-omics web resources for stakeholder communication and strengthened market tactics and behaviours. Moreover, these recent advancements in high-throughput genomics and transcriptomics could enable the development of bio-fortified fruits enriched with macro- and micronutrients. Notably, post-transcriptional gene silencing has been extensively used to prevent post-harvest losses and improve quality (Chen et al. 2020, Sharma et al. 2020a, Yadav et al. 2021). Furthermore, gene editing using CRISPR–Cas9 has emerged as a precise and significant tool of genome manipulation for the development of novel agro-traits in fruits (Kaur et al. 2018, Naim et al. 2018). Fruit markets are highly competitive, requiring producers and sellers to use various strategies to sell desired fruits at reasonable prices. Geographical indication (GI) tags, which indicate unique features and reputations due to their origin in specific geographical conditions, provide intellectual property rights to a product, influencing consumers’ buying behaviour towards GI-tagged fruits (Rajan and Sharma 2018, Datta et al. 2020). In summary, these resources aim to promote sustainable fruit production by integrating molecular breeding and biotechnological intervention to achieve quality, enhanced taste, and increased medicinal values of vital tropical and subtropical fruits. Such efforts aim to establish a robust framework for enhancing tropical and subtropical fruit crop resources in the pursuit of sustainability (Fig. 1).

### Prime tropical and subtropical fruits: origin and distribution

**Figure 2.**
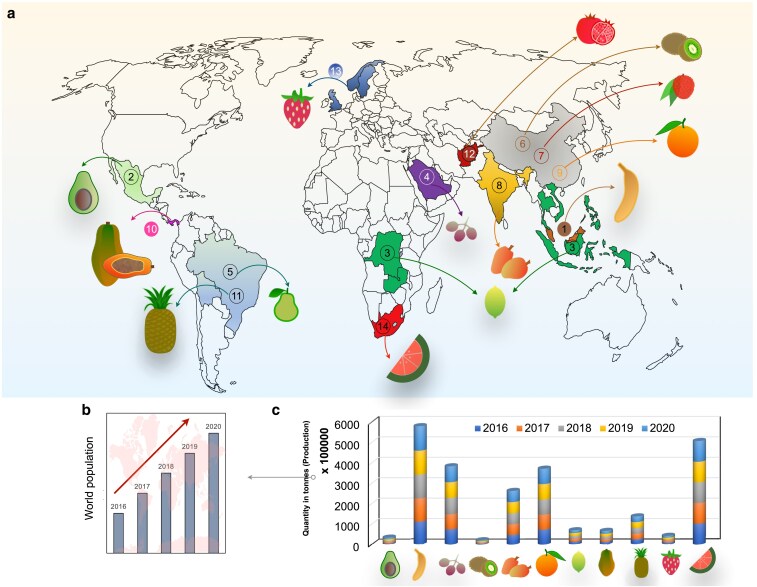
(a) Depiction of the countries of origin for dominant tropical and subtropical fruits on the world map (1) Banana (2), Avocado (3), Citrus (4), Grapes (5), Guava (6), Kiwifruit (7), Lychee (8), Mango (9), Orange (10), Papaya (11), Pineapple (12), Pomegranate (13), Strawberry (14), and Watermelon (b) the exponential increment in world population and (c) Production quantity in x (10 000 tonnes) from 2015 to 2020 for each tropical and subtropical fruit reviewed herein.

The diversity in soil and climatic conditions across the globe provides ample opportunity to grow a large variety of tropical and subtropical fruit species (Mohamed et al. 2011). Citrus, a diverse group of fruits within the family Rutaceae, stands out as a nutraceutical powerhouse rich in phytochemicals and bioactive compounds with remarkable nutritional and health-promoting value. This versatile genus includes economically and nutritionally important fruits such as orange (*Citrus sinensis*), mandarin (*Citrus reticulata*), grapefruit (*Citrus paradisi*), and lime (*Citrus limon*) (Wang et al. 2018a). The Cucurbitaceae, the largest vegetable-producing family, also includes economically important fruit crops such as watermelon (*Citrullus lanatus*) (Maragal et al. 2022). Banana (*Musa acuminata*), originating from Southeast Asia, is an herbaceous flowering plant belonging to the genus Musa and serves as one of the richest sources of dietary energy (D’Hont et al. 2012). A comprehensive description of economically significant tropical and subtropical fruits, their origins, and leading producer countries is provided in (Table 1; Fig. 2). Strawberry (*Fragaria ananassa*), belonging to the Rosaceae family, is widely cultivated for its desirable characteristics, such as colour, texture, odour, and sweetness, making it an excellent commodity for preserved food and nutritional purposes (Ganhão et al. 2019). Grapes (*Vitis vinifera*), belonging to the Vitaceae family, are the world's largest fruit crop, primarily used for wine production, and are a rich source of antioxidants and polyphenols, including flavonoids, tannins, stilbenes, and aroma-producing compounds (Wang et al. 2016b). Pomegranate (*Punica granatum*) is a well-known fruit produced worldwide and belongs to the Punicaceae family. It is a source of many antioxidant substances such as hydrolyzable tannins, condensed tannins, anthocyanins, and organic acids (Nuncio-Jáuregui et al. 2015). Papaya (*Carica papaya)*, belonging to the Caricaceae family, is native to tropical America and is a highly nutritious fruit, enriched with vitamins and harbouring flavonoids, carotenoids, and a myriad of other antioxidant molecules beneficial for consumer health. Additionally, it is rich in vitamins B and C, provitamin A, carotenoids, and a range of phytochemicals such as chymopapain, papain, danielone, protocatechuic acid, caffeic acid, chlorogenic acid, kaempferol, quercetin, p-coumaric acid, oleic acid, linolenic acids, 5,7-dimethoxy-coumarin, carpaine, nicotine, and various proteolytic enzymes (Ali et al. 2014). Mango, a luscious stone fruit obtained from *Mangifera indica*, is a tropical fruit that originated from the Asian subcontinent and is mainly cultivated in India, Indonesia, Myanmar, and Bangladesh (Nuncio-Jáuregui et al. 2015). Guava (*Psidium guajava*), known as the ‘Apple of the Tropics,’ is an excellent source of ascorbic acid and antioxidants, and of high nutritional value (Rodríguez Medina and Valdés-Infante Herrero 2016). Lychee (*Litchi chinensis*), a delicious exotic fruit, is rich in sugar, nutrients, ascorbic acid, and various phytochemicals (Sun et al. 2021b). Avocado (*Persea americana*) is a yellowish-green flesh-rich fruit with a nutty flavour. It is often used in salads or desserts and is a rich source of fatty acids and other vital nutrients (Nath et al. 2022). Pineapple (*Ananas comosus*), from the Bromeliaceae family, is used in many cuisines and contains carbohydrates and proteins. These are a rich source of manganese and ascorbic acid, as well as diverse polyphenols and phytochemicals (Ming et al. 2015). Kiwi fruit (*Actinidia deliciosa*) is an important tropical and subtropical fruit, known for its distinctive and diverse flavour, blending sweetness, tanginess, and subtle fruity notes along with high market demand (Mitra 2014).

**Table 1.** Key economic tropical and subtropical fruits producing countries, their scientific names, origin, leading producer countries, and the data were collected from the official website of Food and Agricultural Organization (FAO), 2024.

Fruits	Scientific name	Origin	Leading producer countries
Avocados	*Persea americana*	America	Mexico, Dominican Republic, Peru
Banana	*Musa acuminata*	South East Asia	India, China mainland, Indonesia
Citrus fruits	*Citrus*	East Asia	China mainland, Spain, Turkey
Grapes	*Vitis vinifera*	Russia	China mainland, Italy, Spain
Guava	*Psidium guajava*	Tropical America	India, Indonesia, China mainland
Kiwifruit	*Actinidia deliciosa*	China	mainland China and Taiwan
Lychee	*Litchi chinensis*	South China	China, India and South Africa
Mango	*Mangifera indica*	India	India, Indonesia, China mainland
Oranges	*Citrus sinensis*	Southern China	Brazil, China mainland, India
Papaya	*Carica papaya*	South Mexico and Costa Rica	India, Dominican Republic, Brazil
Pineapples	*Ananas comosus*	Brazil	Costa Rica, Philippines, Brazil
Pomegranate	*Punica granatum*	Persia	Arabia, Afghanistan, India and China
Strawberry	*Fragaria ananassa*	Northern Europe	Russia, Chile, and the United States
Watermelon	*Citrullus lanatus*	Southern Africa	China mainland, Turkey, India

### Fruit cultivation: global scenario and statistics

The world population is increasing exponentially, and many countries are facing shortages of nutritious food and energy (Irfan et al. 2023). Fruits play a crucial role in agricultural production, and according to the Food and Agriculture Organization (FAO), China, India, Brazil, Turkey, Mexico, and the United States are the leading countries in fruit production. To plan, monitor, and evaluate the development and progress of horticultural products and commodities, various parameters are adopted and streamlined. The first parameter is socioeconomic importance, which encompasses productivity, net profit, resource utilization, land use for production, raw materials, and foreign exchange. The second parameter is the nutritional importance to humanity, focusing on a healthy diet and overall wellness. According to the Statistical Yearbook 2020 released by the FAO, worldwide fruit production is 867,775 thousand tonnes (Singh et al. 2022). The 74th Session of the United Nations General Assembly has declared 2021 as the International Year of Fruits and Vegetables 2021 (IYFV), to raise awareness, directing policy attention, sharing good practices regarding the nutritional and health benefits of fruits, and promoting diversified, balanced, and healthy diets and lifestyles while reducing loss and waste of fruits (del Río-Celestino and Font 2020, Kaparapu et al. 2020). With diversification in consumption patterns, the demand for non-cereal crop cultivation has increased, shifting away from traditional cereal crops such as wheat and rice. This paradigm shift in cultivation patterns has been economically viable and beneficial, leading to higher incomes, urbanization, changing lifestyles, international market integration, and trade liberalization, which have further increased the demand for horticultural products (Jose and Gulati 2023). Strikingly, more than half of tropical and subtropical fruits have been labelled with GI tags to ease their effective market prices and increase specified value potential (Rajan and Sharma 2018, Datta et al. 2020).

### Integration of next-, second-, and third-generation sequencing approaches to fruits

With the progression of next-generation sequencing (NGS) technologies, including second- and third-generation sequencing, significant advancements in genome sequencing of fruits have taken place. Genomics-assisted breeding programmes have made considerable progress due to the increased availability of genomic and transcriptomic sequencing data for various fruit crops (Irfan et al. 2023). The genome sequencing of fruit crops enables researchers to identify linked candidate genes and markers to assign their functions. However, compared to cereals and other economically important crops, genomic studies on fruits have progressed at a slower pace (Chagne 2015). While fruits play a vital role in human nutrition, their significance should also be acknowledged for social upliftment and progress. By integrating recent advances in scientific technology into fruit crops, remarkable outcomes can be achieved in terms of improved growth rates, reduced losses during pre- and post-harvest processing, enhanced quality attributes such as colour, aroma, and firmness, and accelerated breeding for future smart fruit crops. Designing and engineering fruit crops to minimize post-harvest losses poses a challenge for horticulture breeders; however, molecular tools and techniques developed thus far can be deployed for the improvement of smart horticultural crops. Crop genome sequencing projects enhance our understanding of plant biology to enable the development of crops with superior attributes. However, the inherent complexity of genomes, including fruit genomes, can be a barrier to the development of improved varieties.

### Genome sequencing projects: expedite evolution and domestication

A recent breakthrough in second-, third-, and next-generation sequencing is revolutionizing horticultural genomics and accelerating the discovery of fruit traits essential for human health and welfare (Chagne 2015, Heather and Chain 2016). These technologies provide cost-efficient whole-genome sequencing information, which has been an extensively deployed resource for analysing and understanding various functional aspects of fruit crops (Heather and Chain 2016). The grape was the first fruit crop to be sequenced by the combination of 454 and Sanger sequencing, in 2007 by the International Grape Genome Program (IGGP) (Jaillon et al. 2007). The 3× draft genome sequence of the virus-resistant transgenic ‘Sun-Up’ papaya was released by the Hawaii Papaya Genome Project (Ming et al. 2008). In pomegranate, the draft genome revealed selective loci for soft- and hard-seeded populations and identified *MAPK*, *SUC6*, and *SUC8* as possible factors for divergence among tested cultivars (Luo et al. 2020). Pineapple is a widely studied drought-efficient model fruit that possesses facultative crassulacean acid metabolism (CAM), a carbon assimilation pathway that enhances water-use efficiency. Notably, CAM is suggested to have evolved from C3 photosynthesis through the reconfiguration and neofunctionalization of preexisting genes, providing a breakthrough in understanding central metabolic pathways and their evolutionary divergence (Ming et al. 2015). In mango, whole-genome-wide chromosome-scale duplication events and allelic admixture from indigenous varieties exhibit a distinct evolutionary pattern of domestication (Wang et al. 2020b). Surprisingly, whole-genome studies of lychee have revealed two domestication events, through the independent cultivation of early- and late-maturing genotypes (Hu et al. 2022a). Whole-genome analyses of kiwifruit revealed genetic co-linearity, which consequently reflects the genome having gone through two random whole-genome duplications, one genus-specific and the other observed near K–T extinction periods (Wu et al. 2019). Fruit firmness is an essential trait in strawberries that governs post-harvest quality, shelf-life, and end-product qualities. The allo-octoploid heterozygous complex genome has been dissected, and potential QTL governing numerous complex quality traits have been identified (Lee et al. 2021b). Guava, often hailed as the ‘poor man’s apple,’ stands out as a natural reservoir of vitamin C and other essential nutrients. Genomic studies reveal that whole-genome duplication events during domestication reinforced the predominant L-galactose pathway for ascorbate biosynthesis, underpinning its exceptional ascorbic acid content (Feng et al. 2021a). Banana, with a genome size of 523 megabases, is known to have undergone three rounds of duplication in the whole genome of the Musa lineage during the trajectory of domestication and divergence (D’Hont et al. 2012). Whole-genome sequencing of Avocado, a magnoliid, illustrates that there are few translocation events between its homeologous chromosomes despite multiple duplication events (Nath et al. 2022). A chromosome-level alignment provides a clue to interpreting that the sweetness of watermelon arose gradually and was enhanced over the course of domestication, perhaps as the genetic footprints of bitterness were lost during divergence (Renner et al. 2021). Among citrus species, a subset of independent domestication episodes has occurred, resulting in cultivated forms of Mandarin (Wang et al. 2018a). Whole-genome sequencing of tropical and subtropical fruits has provided valuable insights into their genetic diversity and evolutionary relationships, and such information can be leveraged for breeding programmes to improve yield, resilience, and nutritional potential.

### Genomic and transcriptomic resources: mainstreaming growth and developmental events

Genomics and transcriptomic resources serve as a bridge, narrowing the gap between sequence information obtained from various sequencing projects and functional genomics. The resultant transcriptomic signatures are expressive elements associated with biotechnological interventions. Furthermore, these signatures are associated with regulating a plethora of developmental processes, such as ripening, which are important fruit traits but are often overlooked. Detailed information about unigenes and differentially expressed genes under varied tested conditions in a diverse set of selected tropical and subtropical fruits is provided in Table 2. In avocado, the impact of agave fructans on the defence system is revealed via transcriptomics (Cuéllar-Torres et al. 2023) and during asymptomatic infection by avocado sunblotch viroid (Joubert et al. 2024). Transcriptome analysis of mango identified several putative cuticle-associated genes that govern the peel epidermis (Tafolla-Arellano et al. 2017). Transcriptome profiling in mango revealed that *SPL-* and *GA-20-oxidase*-like genes are associated with alternate bearing and carbohydrate metabolic pathways (Sharma et al. 2020b). Moreover, cotyledon development and adventitious root formation are mediated by auxin-regulated proteins, polar transport carriers of auxin, enzymes responsible for cell wall remodeling, and ethylene-related proteins (Li et al. 2017b). Hot water brushing is a commercial method used to improve fruit quality and reduce post-harvest loss. Extensive transcriptomic analysis identified key regulatory genes potentially associated with fruit resistance to biotic stress, lenticel discoloration, and peel colour improvement (Luria et al. 2014). Recent studies suggested differential gene expression is linked to flower development varieties with varying shelf-life (Sharma et al. 2023), identified common and specific cold resistance pathways in both cold-tolerant and non-tolerant varieties (Wang et al. 2024a), and examined how treatment with indole-3-acetic acid can maintain post-harvest fruit quality, antioxidant capacity, and firmness through differential regulation of *Dof1, EIN3, PGs*, and *CX* (Zhou et al. 2023). In guava, a multitude of molecular switches and target candidate transcripts orchestrate fruit development and improve genomic resources for diverse coloured cultivars (Mittal et al. 2020). Magnesium (Mg), an essential element for fruit crops, affects citrus attributes through microtubule movement, cell cycle regulation, signal transduction, and protein phosphorylation (Yang et al. 2019, Ye et al. 2021). Transcriptome and physiological studies on such variegated citrus seedlings revealed the involvement of genes associated with photosynthesis and chlorophyll biosynthesis-related pathways (Xiong et al. 2019). Additionally, transcription factors such as the *bHLH* gene family orchestrate the impacts of salt stress in sweet orange (Zi et al. 2024).

**Table 2.** Dissemination of unigenes (UGNs) and differentially expressed genes (DEGs) for different agronomical useful traits in diverse tropical and subtropical fruits.

Fruits	Traits/Processes	UGN	DEGs	Functional relevance	References
Avocado	Carotenoid biosynthesis	100 837	16 903	Quality progress	(Ge et al. 2019a)
	Ripening processes	151 545	4324	Post-harvest texture	(Liu et al. 2018)
	Diverse ecotypes	154 310		EST-SSR validation	(Ge et al. 2019b)
	Viroid infection		631	Viroid-host interactions	(Joubert et al. 2024)
	Defence system		5425	Extension of shelf-life	(Cuéllar-Torres et al. 2023)
Banana	Benzothiadiazole-responsive regulation		10 313	Elevated defense	(Cheng et al. 2018)
	Dwarfism		4563, 10507	Height regulation	(Cai et al. 2023)
	Ripening physiology		690, 8083, 707, 7403	Post-harvest quality	(Chen et al. 2023a)
	*Fusarium* wilt		172;1856; 800	Host Pathogen interactions	(Tian et al. 2023)
Citrus	Carotenoid synthesis	46 425		Quality enhancement	(Wang et al. 2020a)
	Seedling etiolation	124 952		intuition for skotomorphogenesis	(Xiong et al. 2017)
	Self-incompatibility	50 364		Encourage allogamy	(Ma et al. 2017)
	Seedlessness		106	Consumer preference	(Xiao et al. 2018)
	Huanglongbing		4250	Pathogenesis	(Li et al. 2024c)
Grape	Berry russeting		1491	Insight into russetting	(Huang et al. 2020)
	Ripening process		627	Post-harvest value	(Guo et al. 2016)
	ABA synthesis	52 359		Quality refinement	(Sun et al. 2015b)
	Carotenoid biosynthesis	173		Fortification	(Sun et al. 2015a)
	Interfamily grafting		2113	Genetically distant grafted hybrids	(Zhang et al. 2024a)
	Heat tolerance		3767	Investigate tolerance mechanism	(Wu et al. 2023)
Guava	Fruit Colour		84 206	Augmented tariff	(Mittal et al. 2020)
Kiwifruit	Soil waterlogging	140 187	14 843	Tolerant induction	(Zhang et al. 2015a)
	Sexual phenotype		2503	Insight into development	(Tang et al. 2017)
	Pathogenesis (Bacterial canker)		8255	Insight into resistance	(Wang et al. 2018b)
	Cold-stress tolerance	24 306		Tolerant genotypes	(Sun et al. 2021a)
	Waterlogging	130 246		Mechanism for tolerance	(Li et al. 2022)
	Ascorbic acid metabolism	98 656		Quality refinement	(Liao et al. 2021)
	Ripening process	99 601	28 582	Post-harvest insights	(Tilahun et al. 2020)
	Fruit colouration	100 417		Consumer favoured	(Huang et al. 2021)
	Post-harvest ripening and softening		3116	Mechanistic insights	(Yang et al. 2023)
	Ripening and senescence		2430, 11345	Firmness, ripening	(Wang et al. 2024b)
Lychee	Girdling plus defoliation		2771	Shelf-life improvement	(Li et al. 2015)
	Response to shading	57 050	1039	Fruits induction	(Li et al. 2013)
	Fruit cracking	46 641		Pre-harvest loss	(Li et al. 2014)
	Mg foliar spray response	50 809	1226	Use efficiency	(Wang et al. 2017)
	ROS treatment in rudimentary leaves		5865	Insight into development	(Lu et al. 2014)
	Anthocyanin biosynthesis		9095	Fruit ripening	(Zou et al. 2023)
	Downy blight		36 885	Resistance to lychee downey blight (LDB)	(Yin et al. 2023b)
Mango	Cuticle biogenesis	107 744		Reduce perishability	(Tafolla-Arellano et al. 2017)
	Alternate bearing	12 557		Comprehend mechanism	(Sharma et al. 2020b)
	Adventitious root formation	74 745	1975	Vegetative propagation	(Li et al. 2017b)
	Hot Water brushing		827, 87	Reduction of Post-harvest disease	(Luria et al. 2014)
	Exogenous hormone application		3629, 9121, 3491, 2567, 351	Ripening and senescence	(Zhou et al. 2023)
	Cold stress		8337, 7996, 10683, 10723	Cold resistance	(Wang et al. 2024a)
	Shelf-life		177	Shelf-life improvement	(Sharma et al. 2023)
Orange	Mg deficiency and sufficiency		4864, 4285, 1765	Nutrient use efficient	(Yang et al. 2019, Ye et al. 2021)
	Variegated and green seedlings		4786, 7007	Deduced de-etiolating processes	(Xiong et al. 2019)
	Salt stress		17	Salt-resistant varieties	(Zi et al. 2024 )
	Huanglongbing (HLB)		3604	Pathogenic mechanism of HLB investigated	(Liu et al. 2024a )
Papaya	De-etiolated callus suppression		452	Enlighten photo- morphogenesis	(Jamaluddin et al. 2019)
	Pulp firmness		157	Insight into pulp hardness	(Soares et al. 2021)
	Papaya sticky disease, pre and post flowering		633, 88	Induction for viral response	(Madroñero et al. 2018)
	Ripening mechanism		91	Fruit softening	(Shen et al. 2017)
	Drought responsive	8549	6089	Tissue specific response	(Gamboa-Tuz et al. 2018)
	Water deficit		29, 25	Drought tolerance	(Arroyo-Álvarez et al. 2023)
	Flower development		7159	Flower development and seed germination	(Dai et al. 2023)
	Anthocyanin synthesis		3382, 773, 650	Pericarp colour	(Wu et al. 2024)
Pineapple	White and green leaf cells		1431	Economic gain	(Li et al. 2017a)
	Ethylene-induced floral induction	129 594		Response to growth regulator	(Liu and Fan 2016)
	Pigmentation		1086	Anthocyanin flux	(Zhou et al. 2021a)
	Inflorescence		5370	Induced market cost	(Xie et al. 2021)
	Translucency	24 515	14 426	Quality and shelf-life	(Chen et al. 2023b)
	Flowering induction		271	Floral transition	(Paull et al. 2023)
	Fruit development		25, 17	Bromelain content	(Yow et al. 2023)
	Yellowing and quality		2194	Regulatory pathways in ripening	(Song et al. 2023)
	Anthocyanin biosynthesis		1808, 2859, and 3147	Peel colour determination	(Luan et al. 2023)
Pomegranate	*Xanthomonas* infection	34 626		Resistance breeding	(Singh et al. 2020)
	Scald incidence		952	Inflate commercial shipping value	(Belay et al. 2020)
	Defence for diseases		281	Peel Extract utility	(Belgacem et al. 2019)
	Cold stress		7318	Cold stress	(Liu et al. 2024b)
	Floral development		10373 and 5331	Transitional petal development	(Huo et al. 2023)
Strawberry	Virus infection	36 850	517	Viral resistance	(Chen et al. 2016)
	Fruit development, ripening		6608	Post-harvest management	(Hu et al. 2018)
	Cyanidin metabolism	50 285		Attract consumer	(Xue et al. 2019)
	Anthocyanin pigment	75 426	595	Influence buyer	(Zhang et al. 2015b)
	Botrytis fruit rot		1337, 1265, 1787, 2864	Pathogenesis insights	(Lee et al. 2023)
	Senescence		3861	Ripening and senescence	(Yan et al. 2024)
	ETH and 1-MCP treatment		2346, 833	Role of ethylene in development	(Yu et al. 2024)
	Development and ripening		111	Late embryogenesis abundant (LEA) investigated	(Lin et al. 2024)
Watermelon	Rind colouration	84 516		Consumer attention	(Liu et al. 2020)
	Sugar content		9337	Sugar content	(Zhang et al. 2024b)
	Watermelon mosaic virus (WMV)		616	Pathogenesis factors	(López-Martín et al. 2024)
	Salt stress		3378	Salt-resilient	(Liu et al. 2023)
	Male sterility		1035	Influencing factors identified	(Yue et al. 2023)

These could be leveraged for functional genomics-assisted biotechnological interventions to improve fruits desirable traits.

Bananas, which are nutritionally enriched, often fail to reach their full potential due to *Fusarium* infection. Benzothiadiazole, a compound that induces plant resistance, activates transcription factors, such as *Fusarium* disease-resistant proteins, and plant hormones ethylene and auxin (Cheng et al. 2018, 2019). Race-specific transcriptome analysis in banana suggested marked induction in the expression of pathogen-associated molecular patterns, effector-triggered immunity, ion influx, and pathogenesis-related genes (Cheng et al. 2019). Recent studies on bananas signify plant height regulation through transcriptome and gene co-expression networks (Cai et al. 2023) and further reveal hierarchical gene expression in post-harvest ripening between two diverse banana fruits (Chen et al. 2023b) and response to *Fusarium* wilt (Tian et al. 2023). In papaya, transcriptome studies have been conducted on various agronomical traits, including *DET1* suppression, pulp firmness, pre-flowering stage under sticky disease, insight into the ripening mechanism, and drought response (Shen et al. 2017, Gamboa-Tuz et al. 2018, Madroñero et al. 2018, Jamaluddin et al. 2019, Soares et al. 2021). Recent studies highlight the potential role of *WRKY* and *NAC* transcription factors in papaya during drought tolerance (Arroyo-Álvarez et al. 2023), provide insights into the evolution and divergence of *MIKC*-type MADS-Box genes (Dai et al. 2023), and identify the mechanism behind the formation of purple pericarp via differential expression of many genes, including *MYBs* (Wu et al. 2024). Pineapple has a cluster of differential genes and unigenes associated with white and green leaf cells, floral induction in response to external ethylene stimulation, pigmentation, inflorescence, and translucency (Liu and Fan 2016, Li et al. 2017a, Xie et al. 2021, Zhou et al. 2021a, Chen et al. 2023c). In conjunction, several differentially expressed genes (DEGs), including *HOX21* and *MYB12,* which are specifically regulating the accumulation of anthocyanin in peel (Luan et al. 2023), regulate floral transition (Paull et al. 2023), govern fruit colour and quality (Song et al. 2023), and dissect bromelain enzyme families, which are crucial for protein turnover during developmental cascades (Yow et al. 2023). A multitude of transcripts regulating diverse aspects of growth and development in lychee have been identified through comprehensive transcriptome analyses. These include elements responsive to girdling and defoliation, shading, fruit abscission and dehiscence, foliar magnesium application, and reactive oxygen species (ROS)-induced rudimentary leaf formation (Li et al. 2013, 2014, 2015, Lu et al. 2014, Wang et al. 2017). In addition, coding and noncoding RNAs in the response to downy blight (Yin et al. 2023b) and transcription factor *NAC002* are mediating chlorophyll degradation and anthocyanin biosynthesis by co-activating the expression of both *SGRs* and *MYBs* (Zou et al. 2023). Notably, a few successful transcriptome studies have been performed on pomegranate to test against pathogen attacks, including *Xanthomonas* infection, scald incidence, defence against other severe fruit plant diseases, and the insights on petaloidy and the *BAM* gene family and their role for cold adaptation (Belgacem et al. 2019, Belay et al. 2020, Singh et al. 2020, Huo et al. 2023, Liu et al. 2024c).

In strawberries, infection with strawberry vein banding virus (SVBV) triggers a cascade of gene expression changes, including both upregulation and downregulation of genes involved in pathogenesis (Chen et al. 2016). Similarly, significant transcriptional modulation has been observed, encompassing rapid and substantial changes in genes associated with fruit flavonoid biosynthesis, starch and sucrose metabolism, ubiquitin-conjugating enzymes, and MADS-box transcription factors, all of which contribute to the regulation of ripening processes (Hu et al. 2018). Additionally, the involvement of transcriptomic regulation in cyanidin metabolism in petals of pink-flowered strawberries (Xue et al. 2019), as well as transcript abundance for expressed genes governing red and yellow fruits (Zhang et al. 2015b), has been observed. Recent investigations direct the role of *WRKY29* and *64* in regulating *Botrytis* fruit rot resistance (Lee et al. 2023), the *LEA* family in fruit ripening (Lin et al. 2024), genes governing senescent mechanisms (Yan et al. 2024), and modulation of ethylene signal in regulating sucrose metabolism (Yu et al. 2024). In watermelon, the rind colour, an important agronomic trait affecting market value, is controlled by genes involved in chlorophyll and carotenoid synthesis (Liu et al. 2020). Salt stress significantly reduced DEGs expression related to photosynthesis-related pathways (Liu et al. 2023), was tied to mosaic virus infection (López-Martín et al. 2024), and was associated with male sterility (Yue et al. 2023) and fruit size (Zhang et al. 2024c), opening advanced avenues for novel interventions of immense significance. Transcript profiling for carotenoid synthesis during seed and monocarp developmental stages and ripening processes of avocado has been determined (Liu et al. 2018, Ge et al. 2019a, 2019b). In grapes, lignin and quercetin synthesis are regulatory factors contributing to russeting (Huang et al. 2020), and the transcriptome underlying berry development has been studied (Guo et al. 2016). Comparative transcriptome studies from diverse climates suggest that environmental effects induce berry quality formation (Sun et al. 2015a, 2015b), provide heat tolerance (Wu et al. 2023), and impart interfamily grafting (Zhang et al. 2024b).

In kiwifruits, several transcription factors belonging to the *WRKY, AP2/ERF, MYB, TGA,* and *bZIP* families are potential factors that provide waterlogging responses and tolerance (Zhang et al. 2015a, Li et al. 2022), help dissect distinct sexual phenotypes (Tang et al. 2017), and respond to pathogen encounters, with most of the U-box domain-containing genes being overexpressed (Wang et al. 2018b). Mechanistic insight into kiwifruits shows that *AP2/ERF, MYB,* and *bHLH* transcription factors are involved in cold stress, one of the key abiotic factors (Sun et al. 2021a). Ascorbic acid, a vital vitamin, is synthesized in kiwifruits through three pathways during the process of fruit growth and development (Liao et al. 2021). Ethylene-induced ripening, aimed at improving post-harvest quality, has been evaluated (Tilahun et al. 2020). Additionally, the light response reveals fruit colour through *MYB*-like transcription factors (Huang et al. 2021). Post-harvest quality is a major concern for kiwifruit marketing, but strikingly, ozone treatment can delay the post-harvest softening by regulating cell wall metabolism (Wang et al. 2024d), and treatments of gibberellins inhibit post-harvest ripening, which is imperative for post-harvest market tactics (Yang et al. 2023a). Several crucial transcripts have been identified that dictate carotenoid biosynthesis in the flesh of pummelo, where *HLH* is proposed as an ideal candidate gene controlling carotenoid accumulation (Wang et al. 2020a). A comparative in-depth transcriptomic analysis identified 604 DEGs associated with multicoloured and etiolated seedlings, revealing key transcriptional changes during seedling etiolation (Xiong et al. 2017). In citrus, DEGs governing self-compatibility and incompatibility were characterized to elucidate the genetic basis of seedlessness, a highly desirable agronomic trait (Ma et al. 2017). Furthermore, comparative transcriptomic analyses in mandarin uncovered gene regulatory and signaling pathways associated with female sterility and seedlessness, providing insights into their underlying molecular mechanisms (Xiao et al. 2018). RNA-seq data from huanglongbing reveals disruptions in biological processes within Citrus due to *Candidatus Liberibacter asiaticus* infection (Li et al. 2024c), and a subset of potential responsive events triggered by the same bacterium is revealed via transcriptome sequencing (Liu et al. 2024a).

### QTL: Regulatory cohort for assisted breeding

A QTL refers to DNA sequences associated with discrete phenotypic traits (Fan et al. 2022). These loci may exhibit variable phenotypic expressions, reflecting polygenic behaviour arising from the interaction of multiple genes with their environment (Curtolo et al. 2018, Fan et al. 2022). QTL are often distributed across different chromosomes and are mapped by identifying molecular markers linked to desirable traits, using marker systems such as AFLPs and single nucleotide polymorphisms (SNPs) (Curtolo et al. 2018, Lee et al. 2021a, 2021b, Fan et al. 2022). Fruit breeding relies on these functional marker systems, which associate numerous traits with specific loci (Fan et al. 2022, Nashima et al. 2022). A detailed summary of mapped QTL from studies on tropical and subtropical fruits is provided in Table 3. QTL have been mapped in bananas for traits such as ripening, *Fusarium* wilt resistance, and weevil management (Ahmad et al. 2020, Uwimana et al. 2021, Biabiany et al. 2022). A significant QTL-controlling resistance to the subtropical race of *Fusarium oxysporum* in banana has been reported (Chen et al. 2023a). Extensive approaches have been deployed to screen for many desirable traits in strawberries, including metabolite contents (Vallarino et al. 2019), fruit quality, and flowering (Castro and Lewers 2016). Fruit firmness, an economically significant trait in strawberries, is regulated by *XPA3, PAE8, BGAL5, FLA, ADF1,* and *XTH30F* loci (Lee et al. 2021b). *QSkinCol-1-2, qSkinCol-3-1,* and *qSkinCol-2-3* QTL have been mapped to chromosome 1 for fruit colour, which is a beneficial trait for consumer choice (Castillejo et al. 2020). Major QTL, *FaAAT2, FaGT2,* and *FaOMT* have been recently identified for flavouring traits in strawberries, enhancing their utility (Fan et al. 2022). Genome-wide association studies in a diverse strawberry collection revealed significant marker–trait associations across multiple quantitative trait loci for fruit firmness, and a key breeding target candidate gene, *PG1*, controlling fruit softening and post-harvest shelf-life, was functionally characterized (Muñoz et al. 2024).

**Table 3.** Representation for mapped QTL loci in selected tropical and subtropical fruit crop.

Fruits	Traits	Gene/QTL/Linked gene	Marker type	Chromosome	References
Avocado	Fruit quality	*LG 6, 3, 5*	RAPD, SSR		(Sharon et al. 1998)
Banana	Ripening	*LG 1, LG 7, PM1–7.1*	SNPs	1, 7	(Biabiany et al. 2022)
	*Fusarium* wilt	*Leucine-rich repeat receptor-like kinase*	SNPs	10	(Ahmad et al. 2020)
	Weevil resistance	*Ma05_g24110*, *Ma06_g33600* and *Ma06_g38560*	SNPs	5, 6, 8	(Uwimana et al. 2021)
	Vascular wilt (Foc)	*STR4*	SNPs	3	(Chen et al. 2023a)
Citrus	Aroma volatiles	*FOR1.1, FOR3.4, MUR1.2 and MUR2*	SNPs		(Yu et al. 2017)
	Huanglongbing tolerance	*FS-2015-t6a, FS-2016-t6, CD-2015-t6, CD-2015-t9, etc.*	SNPs		(Huang et al. 2018)
	Seedlessness, flavour and colour	*MUR7.1, MUR7.2, FOR7.2, FOR7.1, FOR4.3, FOR5.1, etc.*	SNPs		(Yu et al. 2016)
	Fruit weight	*FWq3*	SNPs		(Imai et al. 2017)
	Fruit quality traits	Multiple	SNPs		(Imai et al. 2018)
	Fruit quality	*SLG7*	RAPD and SSR		(Curtolo et al. 2018)
	Fruit character	*pds1 and ccd4*	SNPs		(Yu et al. 2016)
	Chloride (Cl^−^) in leaves	*LCl-6*	SCAR	6	(Asins et al. 2023)
Grapes	Berry firmness	*UDV125* and *VMCNG2H2.2*			(Correa et al. 2016)
	Flavonoids accumulation	*VvDFR, VvLDOX* and*VvLAR1*	*eQTL*	Multiple	(Huang et al. 2014)
	Yield components	*Bn4.1; Cn4.1* and *Cn4.2*	AFLP and SSR		(Fanizza et al. 2005)
	Resistance to grey mould	*VlEDR2*	SNP	2, 7	(Su et al. 2023)
	White rot resistance	*PR1*	SNP	3	(Li et al. 2023b)
	Black rot resistance	*Rgb1, Rgb3*	SNP		(Bettinelli et al. 2023)
Guava	Anthocyanin accumulation	*qARI-6-1*	SSRs		(Sohi et al. 2022)
	Softness/hardness	*qFrWt, qSSa, qSSb, qSSc, qSSd*	RAPD		(Padmakar et al. 2016)
	Fruit-quality traits	*C-1, C-2, C-3, C-4, C-5, C-6, C-7*	SNP, InDels		(Maan et al. 2023)
	Colour traits of leaf, peel, and pulp	*qLC1.1, qLL1.1, qLL10.1, qPEA1.2, qPC2.1, qPUA2.1, qPUB10.1, qPUB2.1, qPEB9.1*	SNP		(Mathiazhagan et al. 2024)
Kiwifruit	Russet formation	Multiple	SNPs	3, 11, 19, 24	(Macnee et al. 2021)
	Growth vigour		SNP		(Li et al. 2025)
Litchi	Early ripening	*JHSYH2, JHSYH4, MGL2* and *MGL5*	SRAP and AFLP		(Zhao et al. 2011)
Mango	Fruit weight	*TA1KP1, TA1KP2, TA2KP1, TA2KP2*	SNPs		(Bally et al. 2021)
	Fruit peel colour and firmness	AX-171381971, AX-171379053, AX-171375294, and AX-169929951	SNPs	3, 18, 11, 20	(Srivastav et al. 2023)
	Fruit blush colour, soluble solids	miBCSNP15:10730980, miTSSSNP5:7109828109828	SNPs	15	(Wilkinson et al. 2024)
Papaya	Size and shape	*ovate, sun and fw2.2* homologue	SSRs	Y	(Blas et al. 2012)
	Quantitative traits	*LG1,5, SEX1-1, Q12A-5* and *D2B-1*			(Sondur et al. 1995)
Pineapple	Piping leaf margin	*WOX2, ZFP2, WOX1* and *ZFP CONSTANS*	SNPs		(Sanewski 2022)
	Flesh colour	*AcWOX3*	SNPs		(Nashima et al. 2022)
Pomegranate	Horticultural traits	*PGCT001*	SNPs		(Singh et al. 2015)
Strawberry	Metabolite contents	*FaM6PI1*	SNP and SSR		(Vallarino et al. 2019)
	Fruit quality, flowering	*FxaACAO2I8C-145S, LG IV-S-1*	SSR, SCAR		(Castro and Lewers 2016)
	Fruit firmness	*XPA3, PAE8*, *BGAL5, FLA, ADF1*, *XTH30*FIRM_6-1, 6-4, 3-3 and 5-1	SNPs	3-3, 5-1, 6-1 and 6-4	(Lee et al. 2021b)
	Day-neutrality	*LG IV-T-1*	SSR, SCAR		(Castro and Lewers 2016)
	Fruit colour	*qSkinCol-1-2, qSkinCol-3-1, and qSkinCol-2-3*	SNPs	1	(Castillejo et al. 2020)
	Flavour content	*FaAAT2*, *FaGT2* and *FaOMT*	SNPs		(Fan et al. 2022)
	Fruit firmness	*FaPG1*	SNPs	6	(Fan et al. 2022)
Watermelon	Textural quality	*RH, RTO, RTH and FW*	SNP-CAPS	2, 9, 10	(Yang et al. 2021)
	Sugar traits	*BRX, FRU, SUC, GLU, FWT etc.*	SSRs, in-del, SNPs		(Ren et al. 2014)
	Sugar accumulation	*ClTST2*	SNPs	2	(Ren et al. 2018)
	Gummy stem blight	*ClGSB1.1*, *ClGSB10.1*, and *ClGSB11.1*	SNPs	1	(Hong et al. 2022)
	Gummy stem blight	*ClGSB3.1*, *ClGSB5.1* and *ClGSB7.1*	SNPs	3, 5, 7 and 8	(Gimode et al. 2021)
	Seed weight and coat colour	*q_100SW_2.1, q_100SW_6.1 q_SCC_3.1, q_SCC_5.1* and *q_SCC_5.2*	SNPs	2, 3, 5, 6	(Maragal et al. 2022)
	Gummy stem blight	*Qgsb8.1*	SNPs	8	(Ren et al. 2020)
	Flesh colour	*Cla005011*, *Cla005012*	SNPs	4	(Wang et al. 2019)
	Response for *Fusarium* wilt	*Qfon2.1; Qfon2.2*	SNPs	1, 9	(Ren et al. 2014)
	Gummy stem blight	*qLL8.1*, *qSB8.1* and qSB6.1	SNPs	8, 6	(Lee et al. 2021a)
	Cracking tolerance capacity (CTC), depth of fruit cracking (DFC), rind thickness (RT), and rind hardness	*qCTC-1, qCTC-2, qDFC, qRH, qRT-1, qRT-2*	SNPs	2, 7	(Zhan et al. 2023)
	Fruit size	*fs-B171*	RAD	8	(Li et al. 2023a)
	*Fusarium oxysporum f. sp. niveum (Fon)* wilt resistance			1, 7	(Pal et al. 2023)
	*Powdery mildew resistance*	*ClLox*	dCAPs	2	(Deng et al. 2024)
	Fruit weight	*FW-2-1, FW-5-1, and FW-9-1*	SNP, CAPS	2, 5, 9	(Yang et al. 2023)

These could be utilized for fruit crop breeding and development for socioeconomic importance.

In watermelon, seedlessness, pulp firmness, relative sugar content, and rind colour are desirable consumer traits. QTL, namely *RH, RTO, RTH,* and *FW,* have been investigated in watermelons for textural quality and firmness (Yang et al. 2021), while *BRX, FRU, SUC, GLU, FWT,* and *TST2* have been studied for sugar contents and accumulation (Ren et al. 2014, 2018). A disadvantageous factor for watermelon production is gummy stem blight, caused by *Didymella bryoniae.* Significant QTL for this disease include *qLL8.1, qSB8.1, qSB6.1, Qgsb8.1, ClGSB3.1, ClGSB5.1, ClGSB7.1, ClGSB1.1, ClGSB10.1,* and *ClGSB11.1,* which were mapped to various chromosomes (Ren et al. 2020, Gimode et al. 2021, Lee et al. 2021a, Hong et al. 2022). Molecular mapping of genomic regions influencing fruit and seed morphology in watermelon has been extensively investigated (Yang et al. 2023b). In this context, ClLOX, a QTL, confers resistance to powdery mildew by encoding a lipoxygenase that inhibits pathogen spread and survival (Deng et al. 2024). The gene controlling fruit size was also mapped, revealing a single recessive locus responsible for the small fruit phenotype, with inheritance analyses conducted on novel germplasm and inbred lines exhibiting medium, large, and giant fruits (Li et al. 2023a). Furthermore, QTL-seq was employed to map resistance loci to *Fusarium oxysporum f. sp. niveum* (Fon) race 2, which were subsequently refined to identify key markers for marker-assisted introgression into target loci (Pal et al. 2023). A major QTL with high phenotypic variation was detected on chromosome 2, containing multiple annotated differentially expressed coexisting genes for comparative assessment for fruit cracking (Zhan et al. 2023). Few QTL have been mapped for avocado fruits, which determine fruit quality and cold tolerance (Sharon et al. 1998). QTL governing qualitative and quantitative traits in papaya, including *ovate, sun, fw2.2* homologue, and *LG1, 5, SEX1-1, Q12A-5,* and *D2B-1* for fruit shape and diversity, have been located on different chromosomes (Sondur et al. 1995, Blas et al. 2012). Berry colour and firmness are cost-determining factors in grapes and reflect their quality and post-harvest storage. QTL, *UDV125,* and *VMCNG2H2.2* for firmness (Correa et al. 2016) and *MYBA1* and *MYBA2* for colour development have been established (Sun et al. 2020a). Notably, *DFR* and *LDOX* are the major loci identified as regulating flavonoid accumulation in grapes (Huang et al. 2014), while *Bn4.1, Cn4.1,* and *Cn4.2* signify yield components (Fanizza et al. 2005). Moreover, *Guignardia bidwellii*, the cause of black rot in grapevine, is a challenge for viticulture, and *Rgb3*, a novel QTL, is responsible for 80% of the diversity for bunch resistance, likely by inducing the expression of linked genes for programmed cell death and germin-like protein (Bettinelli et al. 2023). A candidate gene identified as *PR1* is involved in grape white rot resistance, based on QTL detection and analysis, explaining about 17.9% of phenotypic variation (Li et al. 2023b). Two stable QTL related to grey mould resistance were found on linkage groups 2 and 7, with significant phenotypic variance, and a network of structural genes and transcription factors was also involved for grey mould resistance (Su et al. 2023 ). *ANR F1, ANR R1,* and *PGCT001* loci have been established in pomegranate governing anthocyanin content and other horticultural traits (Singh et al. 2015). Dwarf and early-ripening lychee cultivars are desirable for farmers to enhance profitability. Multiple loci, including *JHSYH2, JHSYH4, MGL2,* and *MGL5,* have been established for these traits (Zhao et al. 2011). Similarly, fruit colour and aroma are economically significant traits for which *FOR1.1, FOR3.4, MUR1.2,* and *MUR2* QTL have been identified and established (Yu et al. 2017).

Various QTL have been determined for Huanglongbing tolerance, a devastating disease in citrus (Huang et al. 2018, 2023), including seedlessness (Huang et al. 2018), flavour, and colour (Yu et al. 2016 ), and fruit quality (Imai et al. 2017, Curtolo et al. 2018, Imai et al. 2018). In citrus, a QTL-controlling leaf chloride concentration was found on chromosome 6, with key genes identified as potential transporters for a nitrate and peptide family and facilitator superfamily protein (Asins et al. 2023). Fruit weight, a regulatory trait in mango, has been monitored by *TA1KP1, TA1KP2, TA2KP1,* and *TA2KP2* loci (Bally et al. 2021). Key QTL have also been identified with an integrated genetic linkage map with significant logarithm of the odds scores in at least one tested season for fruit peel colour and firmness, which are major improvement targets (Srivastav et al. 2023). The genetic variation in blush colour is largely explained by highly associated SNPs within the QTL for blush colour and other, related SNPs governing fruit firmness, trunk circumference, and fruit weight are distributed throughout the genome (Wilkinson et al. 2024). *WOX2, ZFP2, WOX1,* and *WOX3* have been found to regulate the piping leaf margin and flesh colour in pineapple (Nashima et al. 2022, Sanewski 2022). In guava, anthocyanin accumulation and fruit softness are attractive traits for consumer choices, regulated by *qARI-6-1, qFrWt, qSSa, qSSb, qSSc,* and *qSSd* loci, along with diverse agronomic traits (Padmakar et al. 2016, Sohi et al. 2022). Quantitative trait loci were found in three environments with best linear unbiased prediction values, affecting 10%–17% of phenotypic variance across multiple environments, suggesting broad stability and utility in guava breeding (Maan et al. 2023). Genotyping and sequencing approaches to develop a high-density linkage map and identify QTL for leaf, peel, and pulp colour open up avenues for understanding colour characteristics (Mathiazhagan et al. 2024). *TPS1a–TPS1d* loci are involved in volatile biosynthesis and other regulatory aspects of growth and development in avocados (Macnee et al. 2021). A high-density genetic map was developed in a diploid kiwifruit population, identifying QTL related to growth vigour and developing single nucleotide polymorphism (SNP) markers for trunk and annual branch diameter (Li et al. 2025). Although breeding remains challenging for tree fruit species, QTLs or trait-linked loci have been identified in tropical and subtropical fruit crops and hold great potential for future breeding programmes. Exploiting these loci can facilitate sustainable fruit production under challenges such as climate change, evolving pathogens, and increasing population pressure, ultimately enhancing social, economic, and health-related outcomes.

### miRNAs: Knowledge-based trait improvements

**Figure 3.**
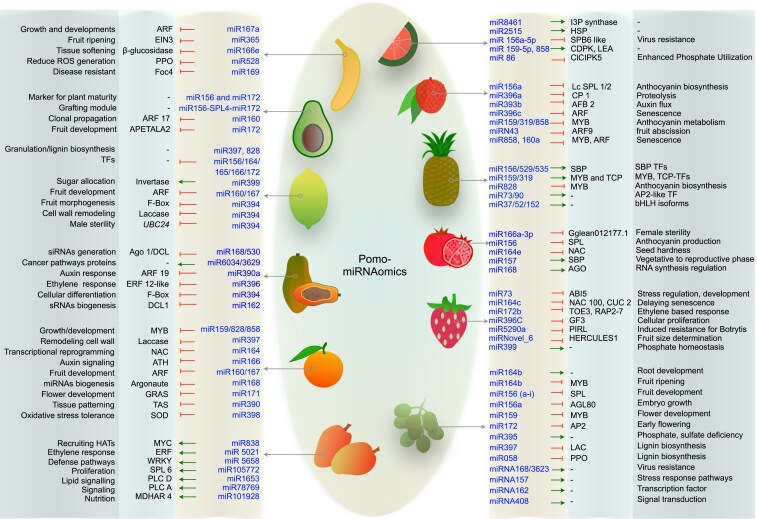
Representation of the potential miRNAs regulating different developmental and metabolic processes in subtropical and tropical fruits. Notation (⊥) represents a functionally validated transcript and downstream effect. A direct green arrow represents possible annotation for particular miRNAs and coherent target genes that have not been functionally validated yet.

miRNAs are small RNA molecules of 19–25 nucleotides that act as essential regulators in plant gene expression and biological processes by serving as evolutionary units that bind to specific target mRNA and regulate post-transcriptional gene expression (Rock 2020, Chandra et al. 2023). In fruits, miRNAs can interact with multiple target genes, influencing important biological processes such as cell growth, tissue differentiation, cell proliferation, and apoptosis (Rock 2020, Sun et al. 2020b). Additionally, competing endogenous RNA acts as decoys for miRNA binding, forming the competing endogenous RNAs (ceRNA) network and regulating the abundance of other RNA transcripts that share the same or similar miRNA response elements (Sun et al. 2020b). miRNAs were identified and characterized in fruits using various computational methods and conventional techniques such as RNA ligase mediated, Poly(A) polymerase mediated-RACE, and qRT-PCR, which validate precursor derivative and expressed sequence tag-derived sequences, as well as target transcript recognition (Dong et al. 2012, Han et al. 2014). Recent literature on fruit studies and developmental cues has grown, but information on functional miRNA-based development has not kept up with genomics-assisted horticulture breeding advancements. A comprehensive list of conserved and novel miRNAs that regulate agronomic traits in tropical and subtropical fruits is depicted in (Fig. 3, Table 4). In avocado, genome-wide analysis revealed that miRNA160 and its cognate target *ARFs* are involved in somatic embryogenesis and may contribute to the low efficiency of embryo induction (Lorenzo-Manzanarez et al. 2025). *Piriformospora indica* is a mycorrhizal fungus renowned for providing plant resilience to *Fusarium* wilt in bananas by regulating the expression of key genes and transcription factors regulated through miRNAs, thereby enhancing the plant's defensive capabilities (Wang et al. 2024b). A frequent negative correlation between the expression of miRNAs and target host genes in *Musa acuminata* during interaction with *Pseudocercospora musae*, a causal agent of Sigatoka leaf spot disease (Rego et al. 2023). miRNAs and gene expression related to *ACC oxidase* developed via RNA interference-based transgenic bananas, revealing pathways enriched in the sulphur relay system and starch and sucrose metabolism (Xia et al. 2023). Transcriptional regulation of the miRNA528–*PPO* module by miRNA156-targeted *SPLs* orchestrates the chilling response in banana, providing evidence for crosstalk between transcription factors and miRNAs (Kong et al. 2025). miRNAs have been postulated to be part of diverse grape genotypes and play a vital role in anthocyanin and flavonol accumulation via modulating *MYB* (Chen et al. 2019, Tirumalai et al. 2019). These small guiding stretches maintain signal transduction pathways for seed and stone-hardening regulatory networks (Wang et al. 2021a). The miRNA156: *SPB* module has been well-described and is involved in the regulation of grape berry growth and ripening (Wang et al. 2016a), and in regulating various aspects of growth and development processes (Wang et al. 2013, 2014, Leng et al. 2015). A set of cold- and high-temperature-responsive miRNAs and their targets in grapevine, revealing that these miRNAs target and control the expression of transcription factors like *ARF*, *SPL*, and *GRAS* to adapt to corresponding stresses (Rooy et al. 2023, Zhang et al. 2024a), and the functionality of miRNA167 is confirmed through ectopic transformation, revealing enhanced heat tolerance in transgenic lines by positively regulating thermostability (Zhang et al. 2023b ). Strikingly, the grafting of rootstock to grapevine leaves increased the resveratrol content of the scion by downregulating the expression of miRNA171c. This new target could be a promising avenue for improving plant and human health via miRNA engineering (Zhu et al. 2024a). Granulation in citrus juice sacs leads to post-harvest losses in the entire citrus family, and miRNA families, such as miRNA397 and miRNA828, targeting *Cs6g06890.1* and *Cs1g17590.1*, respectively, could regulate sac granulation processes by impacting inner lignin metabolism (Zhang et al. 2016). Mining of miRNAs from expressed sequence tag (EST) sequences has been reported in the citrus family (Song et al. 2010); however, their functional roles were characterized in Mexican lime during early and late stages of Huanglongbing, with target proteins predominantly comprising transcription factors broadly involved in the disease’s symptomatic progression (Bojórquez-Orozco et al. 2023). Recently identified miRNA171b positively regulates resistance to Huanglongbing by targeting and downregulating the expression of *SCARECROW*-like genes (Lv et al. 2023). Recently, Hu et al. (2022b) reported that miRNA399-3p and its target gene, neutral/alkaline invertase, are significant modulators that determine citrus fruit quality and regulate scion-rootstock interaction. Exosome-like nanoparticles from *Citrus reticulata* juice were found to suppress *Penicillium* growth, with miRNA-like sequences playing a key role in their inhibitory activity, providing strong evidence of citrus fruit-derived cross-kingdom RNAi-mediated regulation (Yin et al. 2023a). In conjunction, not surprisingly, cross-kingdom analysis has revealed that fruit-derived miRNAs, apart from antioxidants, can block the activity of viral RNA by targeting the UTRs (untranslated region) and can become potent therapeutic candidates for viral genome silencing, especially against SARS-CoV-2 and its havoc variants (Mangukia et al. 2021, 2022). Beyond their roles in plant biology, papaya miRNAs are also computationally predicted to target genes implicated in human cancer pathways, highlighting their potential in cross-kingdom gene regulation. However, the lack of functional validation currently limits their translational and therapeutic applications (Jha et al. 2021). In silico identification of papaya genome-encoded miRNAs targeting begomovirus genes responsible for papaya leaf curl disease has been reported; however, their functional potential remains largely unexplored in the context of disease resistance mechanisms (Srivastava et al. 2024). Pineapple's spatial-temporal miRNA dynamics are a key feature of obligate Crassulacean acid metabolism plants, making it a potential candidate for developing drought-tolerant fruit crops (Bai et al. 2019), and the involvement of miRNAs in pineapple anthocyanin biosynthesis, photosynthesis, and ethylene-triggered flowering in related families has been delineated (Wai et al. 2017, Ding et al. 2020, Rock 2020). miRNAs computationally predicted in mango are temperature-responsive (Moh et al. 2021), based on expressed sequence tags (Yadav et al. 2021). However, according to the available information, limited miRNA targets have been functionally validated in this delicious fruit. Strikingly, the elucidation of miRNAs and their putative targets associated with jelly seed disorder in mango opens new avenues for improving key consumer satisfaction traits (Ahmad et al. 2024). In watermelon, miRNAs have been characterized that are responsive to the cucumber green mottle mosaic virus (Sun et al. 2017), and ceRNA networks involved in disease response have been developed (Sun et al. 2020b).

**Table 4.** Proclamation of known and novel miRNAs and their predicted target transcript in assorted beneficial traits in diverse tropical and subtropical fruit species.

Fruits	Test condition	Novel miRNAs	Known miRNAs	Number of predicted targets	References
Banana	*Fusarium oxysporum* (Foc) responsive	28	254		(Cheng et al. 2019)
	Fruit ripening	5	2		(Xia et al. 2023)
	Sigatoka leaf spot disease	24	30	11	(Rego et al. 2023)
	*Fusarium* wilt	51, 54	69, 24		(Wang et al. 2024b)
Citrus	Cell wall remodeling	10	42	4	(Song et al. 2010)
	Agronomical traits	10	63	4	(Song et al. 2010)
	Citrus granulation	12			(Zhang et al. 2016)
	Pathogenic infection	4	96		(Yin et al. 2023a)
	Huanglongbing disease	17	29		(Bojórquez-Orozco et al. 2023)
Grapes	Berry development	132	242	193	(Wang et al. 2014)
	Cold-inducible	67	163	13	(Sun et al. 2015b)
	Development and fruit quality	20	177		(Zhang et al. 2024a)
	Cold stress	181	158		(Rooy et al. 2023)
	Heat stress	86	873		(Zhang et al. 2023b)
	Environmental stress	267	177		(Zhu et al. 2024a)
Guava	Salinity stress	40		49	(Sharma et al. 2020a)
Mango	Temperature responsive	104		2347	(Ding et al. 2020)
	Stress responsive		18	44	(Yadav et al. 2021)
Orange	Development specific	27, 10	39, 42		(Song et al. 2010)
Pome-granate	Female sterility	58	103	144	(Chen et al. 2020)
	Pistil development	348	61	4952 and 6932	(Zhao et al. 2024)
Strawberry	*PHAS* loci and miRNAs annotation	54	167		(Li et al. 2016)
Watermelon	Cucumber green mottle mosaic virus	1809	471		(Sun et al. 2017)
	Male fertile and sterile floral buds	285	29		(Zhang et al. 2024b)

Moreover, miRNAs have been screened and detected for their responses to *Fusarium* wilt and for regulating various biological and metabolic processes in watermelons (Wang et al. 2024c, 2025a). Interestingly, melatonin-induced downregulation of miRNAs, including miRNA159-5p, miRNA858, miRNA8029-3p, and novel-m0048-3p, is accompanied by upregulation of their corresponding target genes involved in signal transduction, thereby enhancing protection under cold-stress conditions (Li et al. 2016). miRNA-assisted grafting is widely utilized to improve watermelon by enhancing nutrient utilization and stress tolerance (Wu et al. 2021). Furthermore, (Feng et al. 2019) speculated on the integrated functional aspect of miRNA and *PHAS* loci in strawberries. Assorted *PHAS* loci were targeted by two different miRNAs, with one target site triggering phasiRNA generation and another providing targeting sites for phasiRNA generation, subsequently regulating divergent cellular cascades. Additionally, the functional regulation of anthocyanin biosynthesis was demonstrated by miRNA858 in kiwifruit through the targeting of *MYBC1* expression, revealing its potent effect on fruit growth and development (Li et al. 2020). Moreover, miRNA160d regulates plant disease resistance against *Botrytis* infestation through the auxin signaling pathway, highlighting the potential of these small RNAs as valuable resources for enhancing disease resistance (Li et al. 2023c). Conversely, two members of the miR482 families, miR-215-3p and miR-29-3p, were found to increase kiwifruit sensitivity to *Pseudomonas* when overexpressed (Jiang et al. 2024). From an evolutionary perspective, the loss of miRNA targets following gene duplication contributes to expression divergence among gene duplicates, thereby stabilizing gene expression within miRNA-targeted networks and leading to variable citrate accumulation (Ling et al. 2024). Differentially expressed miRNAs in male sterile and fertile watermelon floral buds have been identified, and their target genes potentially involved in biological processes like floral organ development and pollen maturation were established (Zhang et al. 2024). miRNA156 and 166 played a crucial role in regulating the development of pomegranate pistils by negatively influencing cellular homeostasis (Zhao et al. 2024). While miRNA-mediated regulation in major tropical and subtropical fruits is well documented, knowledge of transcription factor binding motifs in these crops remains scarce, limiting the development of comprehensive gene regulation models and highlighting the need for further investigation. By elucidating the functional mechanisms through which miRNAs control agronomical traits, researchers can develop strategies for improving desirable traits, which offers significant potential for a sustainable fruit industry.

### Gene editing: accelerating the potential of genomic resources

**Figure 4.**
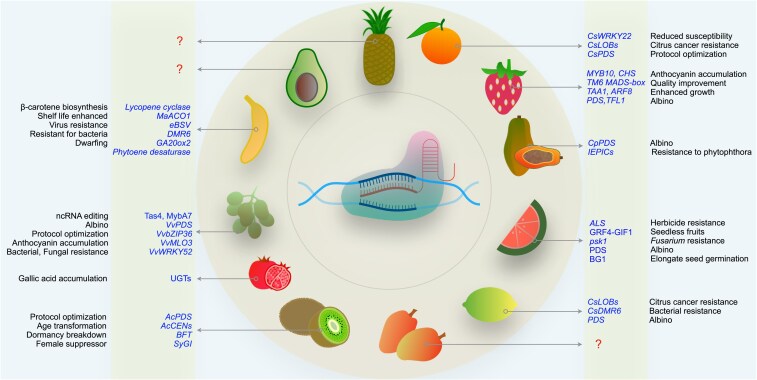
Schematic showing the demonstrated application of CRISPR/Cas9 in trait rediscovery and generating new traits by editing genes of interest for biological significance in tropical and subtropical fruits.

The advancements in gene editing technologies have revolutionized the fields of functional genomics and crop improvement. Programmable nucleases have provided researchers with the ability to manipulate any genomic sequence virtually. Conventional CRISPR/Cas9 tools and their diverse modifications yielded many attractive applications, such as CRISPRa for gene activation, CRISPRi for gene interference, base editors for introducing point mutations, and prime editors for installing predefined insertions or deletions (in-dels) in the genome (Kaur et al. 2018, Karmakar et al. 2022b). These tools have been widely adopted for genome editing in multiple tropical and subtropical fruit crops. In most cases, the feasibility of genome editing has been demonstrated by targeting the *phytoene desaturase* (*PDS*) gene, where knockout mutations result in albino phenotypes. For example, *PDS* was knocked out in kiwifruit (Wang et al. 2018d), papayas (Brewer and Chambers 2022, Elias et al. 2023), bananas (Kaur et al. 2018, Naim et al. 2018), citrus (Dutt et al. 2020), watermelons (Tian et al. 2017), strawberries (Wilson et al. 2019), and grapes (Ren et al. 2021) (Fig. 4). A mesophyll protoplast-based system was developed for efficient gene editing in papaya, targeting *PDS* and *MLO6* genes to generate knockouts in different cultivars (Elias et al. 2023). In bananas, editing the ortholog of *DMR6* provides enhanced resistance to the bacterial disease (Tripathi et al. 2021), while *ACO1* (*aminocyclopropane-1-carboxylate oxidase 1*) promotes extended shelf-life via reduced ethylene synthesis (Hu et al. 2021). Tall banana plants frequently experience lodging due to frequent high winds and storms, causing huge losses to farmers. To overcome this problem, a semi-dwarf banana was generated by editing the *GA20ox2* gene (Shao et al. 2020). Importantly, biofortification has emerged as a research focus to combat hidden hunger and malnutrition, and fruits are particularly compelling candidates for biofortification. Therefore, gene editing has been employed in fruits to enhance their nutritional value. For instance, editing of lycopene epsilon-cyclase has resulted in improved carotene biosynthesis in bananas (Kaur et al. 2020). Grapes have undergone large-scale editing to uncover functional genes associated with various desirable traits (Wang et al. 2016b). Furthermore, successful editing of the *IdnDH* (*L-idonate dehydrogenase*) gene in ‘Chardonnay’ suspension cells was achieved, followed by the generation of plantlets (Ren et al. 2016).

CRISPR/Cas9-mediated removal of one allele of *ZIP36* promoted anthocyanin accumulation in grapes through negative feedback (Tu et al. 2022). In pomegranate, editing using gRNAs designed against UDP-dependent glycosyltransferases (*UGTs*) resulted in distinct accumulation of gallic acid, facilitating germplasm improvement (Chang et al. 2019). Being sessile, fruit trees are constantly exposed to various biotic factors such as bacteria, fungi, viruses, and more. Genome editing has been successfully applied to diverse fruit crops to develop resistance against these pathogens (Karmakar et al. 2022b). The B genome of plantain banana (AAB) contains integrated endogenous banana streak virus (*eBSV*), which poses significant challenges in breeding and hybrid dissemination. CRISPR/Cas9-mediated disruption of the virus sequence has successfully deactivated the endogenous *eBSV* (Tripathi et al. 2019). Powdery mildew, a devastating disease of grapevines causing significant yield loss, has been combated by enhancing resistance through CRISPR/Cas9-mediated editing of *MLO3*, a host susceptibility gene (Wan et al. 2020). Similarly, editing of the *WRKY52* gene in grapes has restored resistance to fungal encounters (Wang et al. 2018c). Additionally, the *TAS4b* and *MYBA7* genes have been edited in grapes using CRISPR/Cas9 technology to generate bacterial and viral resistance, respectively (Sunitha and Rock 2020). Likewise, genome editing has been successfully employed to develop resistance to bacteria in citrus (Jia et al. 2017, Wang et al. 2019), for fungus in watermelon (Zhang et al. 2020), and against bacteria in banana (Hu et al. 2021). A set of edited lines with downregulation of *NPR3* was generated successfully, providing stable editing opportunities for citrus intervention (Mahmoud et al. 2022). CRISPR diagnostics could also be successfully used for the early detection of pathogens in fruit trees (Karmakar et al. 2022a).

In kiwifruit, editing the *BFT2* gene has shown potential in reducing plant dormancy without adversely affecting flowering (Herath et al. 2022). Repressing flowering using *CENTRORADIALIS-like* genes has enabled the transformation of perennial woody kiwifruit into rapid terminal flowering, achieving the transition from perennial to annual growth habit (Varkonyi-Gasic et al. 2019). Efficient editing of *CEN, CEN4,* and *SyGl* genes has been achieved using CRISPR/Cas9, mutagenizing male kiwifruit (Varkonyi-Gasic et al. 2019, De Mori et al. 2020). Integration of a *GRF4-GIF1* cassette with CRISPR-associated protein has facilitated highly coordinated gene editing, leading to the generation of seedless watermelon (Feng et al. 2021b). CRISPR/Cas9 was used to knock out the *eIF4E* gene in watermelon, resulting in a truncated protein and developmental defects in plant growth, leaf morphology, and reduced yield (Li et al. 2024b). In strawberries, the *TM6 MADS*-box gene has been edited using CRISPR/Cas9, opening new avenues for octoploid species (Martín-Pizarro et al. 2019). Ten lines of *PG1* knockout strawberry plants were generated using CRISPR/Cas9, enhancing strawberry fruit firmness, a desirable trait for consumer preference (López-Casado et al. 2023). A CRISPR/Cas9-targeted knockout of *PHO_2_* can enhance the phosphorus content and improve the fruit quality of woodland strawberries (Zhang et al. 2023a). CRISPR-mutated *β-glucosidases* (*BG1*) have been shown to stimulate seed germination and reduce seed size in watermelons (Wang et al. 2021b). Base editing is a precise technique derived from CRISPR/Cas9, enabling the alteration of a single nucleotide in the genome (Molla et al. 2021). Using base editing, herbicide-resistant watermelon has been generated by targeting the *acetolactate synthase* (*ALS*) gene (Tian et al. 2017). Furthermore, base editing has also been employed to modify the sugar content of strawberries (Xing et al. 2020). The availability of numerous successful examples should encourage the utilization of the CRISPR/Cas9 system for functional genomics and the improvement of agronomic traits in other tropical and subtropical fruits. Prominent fruits such as mangoes, pineapple, and avocados remain largely unexplored in the context of gene editing. Overall, this advancing technology holds great potential to enhance fruit taste and flavour, as well as reduce post-harvest losses.

### Web genomics resources: a platform for stakeholder communications

**Figure 5.**
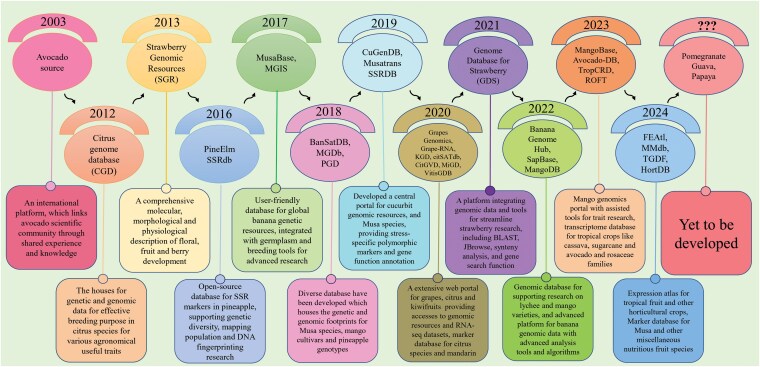
A chronological timeline of events in the development of web databases hosting genomic resources for tropical and subtropical fruit crops. These developed web resources have tremendous potential to transform the fruit crops market potential with proper utilization of the wealth of information they host. All the figures presented in this review have been created using integrated tools such as BioRender (https://www.biorender.com/), Canva (https://www.canva.com/en_in/), and PowerPoint.

Web genomic resources encompass a variety of computational tools, databases, portals, and web servers that provide convenient access to varietal databases containing genomic, transcriptomic, and proteomic information for various agronomically advantageous traits in fruit crops (Li et al. 2024d, Roy et al. 2024). Recently, HortDB was developed, aiming to be the leading platform providing omics and breeding data to facilitate breeding (Li et al. 2024d). In connection with tropical and subtropical fruits, various web-based resources are designed to propagate fruit crop variation and expression atlases of functional footprints (Xiao et al. 2023, Liu et al. 2024b, Roy et al. 2024). Several important centralized repositories, such as the Genome Database for Rosaceae (GDR) and the Rosaceae Fruit Transcriptome Database (ROFT), houses data for fruits such as apples, pears, raspberries/blackberries, strawberries, and other stone fruits and exclusively include diverse genetics and genomics data, featuring annotated EST sequences, markers, traits, and genetic, physical and transcriptomic maps (Jung et al. 2019, Li et al. 2023a). MusatransSSRDB and the Musa marker database provide a comprehensive genomic resource to facilitate effective banana breeding (Backiyarani et al. 2019, Biswas et al. 2024). MGIS and the Banana Genome Hubs are an alternative resource used for managing genetic information and high-throughput genotyping data for novel trait discoveries (Ruas et al. 2017, Droc et al. 2022). A detailed chronological description of the developed databases on the discussed fruits has been provided in (Fig. 5). Strawberry Genomic Resources (SGR) and Genomic Database for Strawberries (GDS) provide a repository for descriptions, sample statistics, gene annotations, gene expression analysis, gene function information, and gene ontology (GO) assignments. The purpose is to facilitate the dissemination and data mining of extensive floral and fruit transcriptome data in the woodland strawberry (Darwish et al. 2013, Zhou et al. 2021b). The Citrus Genome Database and Mikan Genome Database have been developed to populate citrus genome sequence resources and tools, offering molecular marker mining tools for breeding programmes (Gmitter et al. 2012, Kawahara et al. 2020). The Cucurbit Genomics Database, developed using the Tripal toolkit, incorporates genome and EST sequences, genetic maps, and transcriptome profiles for cucurbit species, as well as sequence annotations, biochemical pathways, and comparative genomic analysis. These include synteny blocks and homologous gene pairs among different cucurbit species, along with analysis and visualization tools for viewing synteny (Zheng et al. 2019). SapBase is a comprehensive platform offering access to functional and comparative genomics data in lychee, as well as several other fruit species (Li et al. 2024a).

The Vitis International Variety Catalogue (VIVC) serves as an information source for breeders, researchers, and curators of germplasm repositories. It provides SSR-marker data, a comprehensive bibliography, and illustrations (Maul and Töpfer 2015). Notably, VitisGDB offers information for grapevine-assisted breeding and intervention (Dong et al. 2020). The mango SNP database contains SNPs obtained from the restriction site-associated DNA (RAD) sequence of different cultivars. It serves as a genomic resource for mapping, QTL studies, genome finishing, molecular breeding, phylogeographic and evolutionary studies (Iquebal et al. 2017). Few other databases provide access for mango varieties and genomics expression atlas (Radha et al. 2018, Gómez-Ollé et al. 2023). The Kiwifruit Genome Database (KGD) contains publicly available genome and gene sequences, gene annotations, biochemical pathways, transcriptome profiles derived from public RNA-Seq datasets, and comparative genomic analysis results, including syntenic blocks and homologous gene pairs between different kiwifruit genome assemblies (Yue et al. 2020). PineElm_SSRdb is a microsatellite marker database derived from pineapple genomic, organelle, and EST sequences that provides avenues for cross-species transferability of markers and investigating diversity (Chaudhary et al. 2016). At present, there is a lack of dedicated databases for fruit crops such as pomegranate, papaya, and guava.

### Single-cell sequencing: unlocking cellular heterogeneities

With increasing interest in the nutrient richness and health-promoting attributes of tropical and subtropical fruits, developing elite genotypes with desirable traits has become a top priority. However, often cellular complexity and molecular heterogeneity hinder such progress in both breeding and applied interventions. Recently, the advent of single-cell sequencing has opened an unprecedented window of opportunity for horticulturists, enabling the dissection of gene expression dynamics at single-cell resolution across precise developmental stages and stress conditions (Bai et al. 2022, Yang et al. 2023). Despite limited application in tropical and subtropical fruit species, single-cell omics has already demonstrated its transformative potential. In woodland strawberry, single-cell transcriptomic profiling during *Botrytis* infection revealed distinct cell populations and their coordinated defence responses. Key defence-associated transcription factors, such as *WRKY75* and *NAC042*, were highly expressed in specific epidermal and mesophyll cells, orchestrating the transition from growth to defence and activating systemic immunity (Bai et al. 2022). Similarly, single-nucleus RNA sequencing in lychee apical buds uncovered a surprising lack of *FT* and *TFL1* gene expression in bud nuclei, despite bench experiments confirming their presence, highlighted the central role of *TFL1-2* in repressing flowering, and proposed that the *FT1/TFL1-2* expression ratio may serve as a molecular switch governing floral transition (Yang et al. 2023). Although many of the discussed tropical and subtropical fruit crops have yet to benefit from single-cell omics studies, the compelling evidence from these pioneering studies strongly advocates for the broader implementation of this approach. Harnessing such cutting-edge tools and technologies will be critical for decoding complex trait architectures and accelerating breakthroughs in fruit crop improvement.

### Multi-omics integration and resources: unveiling complex traits

Multi-omics studies are essential in tropical and subtropical fruits for deciphering complex traits, enhancing nutritional quality, and improving stress resilience. In avocado, an integrated multi-omics analysis revealed a desynchronization between colour change and fruit softening (Nunez-Lillo et al. 2023). Additionally, multi-omics approaches have been employed to visualize ecotype-specific metabolite heterogeneity in mesocarp tissues (Ge et al. 2021) and to elucidate rootstock–scion interactions (Núñez-Lillo et al. 2024). In bananas, multi-omics analyses during ripening identified differentially expressed genes, accumulated proteins, and metabolites in the peel, with key transcription factors primarily from the *ERF* and *bHLH* families (Yun et al. 2019). Moreover, inhibition of flavone and flavonol accumulation at low post-harvest temperatures was found to be mediated by downregulation of *bHLH* transcription factors (Song et al. 2024). In citrus, multi-omics approaches have uncovered the involvement of various structural genes and key transcription factors in flavonoid biosynthesis. A significant decline in the expression of genes such as *CHS, CHI, FLS, F3H,* and *4′OMT* during fruit development was observed, correlating with the reduction in flavonoid metabolite accumulation (Zhu et al. 2024b). Moreover, multi-omics approaches have provided critical insights into citrus biology and the underlying mechanisms of fruit quality traits. These include the elucidation of polymethoxylated flavonoid biosynthesis pathways (Wen et al. 2024), the identification of causal variations underlying bioactive flavonoid content across diverse genotypes (Huang et al. 2025), and the discovery of key regulatory factors involved in citrus fragrance formation through the study of glossy mutants (Wan et al. 2022). Additionally, the importance of chromoplast plastoglobules in carotenoid accumulation has been highlighted (Liu et al. 2024), along with citrus responses to Huanglongbing infection (Chin et al. 2021). A functional gene encoding flavanone 3-hydroxylase (*F3H*) has been identified, wherein natural variation in this gene contributes to differences in dihydrokaempferol levels, driven by genetic polymorphisms in both promoter and coding regions (Mou et al. 2021). Multi-omics analysis has also revealed genetic differences among valuable genotypes of various citrus, which are widely used as medicinal herbs (Wang et al. 2025b).

In the glossy orange mutants, sesquiterpenoids, particularly valencene and caryophyllene, accumulated at significantly higher levels compared to wild-type fruits. Interestingly, upregulated metabolites in the mutants showed a strong positive correlation with valencene levels during fruit maturation, whereas downregulated metabolites exhibited a negative correlation (Wan et al. 2022). Furthermore, a comprehensive multi-omics study identified *AAT1,* an alcohol acyltransferase, as a key gene responsible for ester production contributing to orange flavour (Fan et al. 2023), and the candidate gene mapping further delineated loci associated with aroma traits (Hu et al. 2024). In grapes, water deficit triggers both ABA-dependent and ABA-independent signal transduction pathways by modulating the expression of multiple transcription factors. Multi-omics network analyses have revealed that drought-responsive transcription factors, including *bZIPs, AP2/ERFs, MYBs*, and *NACs*, play key roles in regulating stress-associated metabolites (Savoi et al. 2017). Integrated multi-omics approaches have also elucidated the biochemical basis of aroma diversity across multiple grape genotypes. Distinct aromatic profiles were found to be significantly influenced by variations in terpenoids, heterocyclic compounds, and ester compounds regulated by key enzymes such as *CXE, TPS, LOX,* and *AAT* (Xun et al. 2025). Additionally, developmental stage-specific metabolic profiling has provided insights into the temporal dynamics of metabolite accumulation (Sampaio et al. 2024). Moreover, UV-C irradiation was shown to selectively induce the stilbene biosynthetic pathway, shaping the metabolic profile of berry skin ideotypes (Suzuki et al. 2015). In kiwifruit, spatiotemporal regulatory networks have been constructed, leading to the functional characterization of transcription factors such as ethylene response factor 1 (*ERF1*), basic leucine zipper 60 (*bZIP60*), and *RAP2.4*, all of which are involved in various molecular and developmental processes (Zeng et al. 2025). The regulatory impacts of ethylene and 1-methylcyclopropene treatments on shelf-life and fruit quality have also been assessed (Li et al. 2024e), resulting in the identification of key post-harvest biomarkers of fruit maturity such as *TIP4-1, MYB10,* and *β-amylase,* which were recognized as the most reliable indicators (Favre et al. 2023). More integrated studies also shed light on the molecular mechanisms underlying unique flavour development in kiwifruit (Wang et al. 2022). In guava, multi-omics analyses have unravelled the complex regulatory networks governing fruit ripening, highlighting several metabolic pathways and growth regulators that influence ripening progression. Notably, anthocyanins, the end products of the phenylpropanoid pathway, were found to be synthesized during ripening, contributing to pigmentation and nutritional quality (Monribot-Villanueva et al. 2022). In strawberry, integration of regulatory elements with Genome-wide association studies-linked allele-specific gene expression has uncovered genetic variations associated with volatile compound biosynthesis, which are critical for flavour development (Fan et al. 2022). The functional role of anthranilate synthase in this process was further validated through transient fruit expression assays. In watermelon, a unique combination of fine-scale multi-omics genetic mapping and transcriptomic analysis led to the identification of a major gene responsible for the distinct, clear stripe margin pattern on the peel (Yang et al. 2024). These findings demonstrate the power of multi-omics integration as a promising approach to unravel the molecular basis of complex traits in tropical and subtropical fruits. Future research should aim to elucidate the molecular mechanisms underlying intricate phenotypic traits and prioritize the use of multi-modal omics strategies to dissect regulatory networks, with the ultimate goal of enhancing agronomic performance and fruit quality.

High-value insights from multi-omics research require efficient management and dissemination to ensure their practical utility for breeders and scientists. To address this need, multi-omics web databases have been developed to provide information for unlocking genetic potential and promoting climate-smart horticulture in tropical and subtropical crops, which exhibit high data complexity and biological diversity. For instance, the recently developed TropCRD (Xiao et al. 2023a) provides a comprehensive system for collecting and analysing variation information in economically important tropical crops. TCOD (Kang et al. 2024) integrates diverse omics datasets across multiple species, serving as a platform for cross-species comparisons through homology-based connections. Similarly, GERDH (Cheng et al. 2023) is an interactive multi-omics database designed for horticultural crops, enabling large-scale, cross-species data mining and enhancing accessibility to omics data, particularly in fruits. Therefore, there is a pressing need to develop more integrated, user-friendly, and crop-specific databases for other tropical and subtropical fruits to bridge the current gaps in multi-omics data dissemination and to realize the potential of these valuable resources.

### Conclusion and challenges

The genetic and genomic resources of tropical and subtropical fruits provide a critical foundation for advancing breeding programmes and horticultural interventions. Despite considerable progress in sequencing technologies and molecular characterization, comprehensive genomic data and well-annotated genomes of key tropical and subtropical fruit species are still lacking. Such data are essential to streamline various downstream applications. However, the high genetic diversity, heterozygosity, polyploidy, and large genome sizes characteristic of some tropical fruit species pose significant challenges to these efforts. Emerging technologies, such as long-read sequencing and single-cell genomics, hold the potential to address these challenges and revolutionize fruit genomics. Transcriptomic analyses have identified key genes associated with advantageous agronomic traits. However, functional insights into gene regulation remain a critical limitation for future interventions. A more mechanistic understanding of gene function and regulation will be crucial for guiding future research in this area. QTL mapping and genetic studies linking specific genes to traits of interest have proven effective in enhancing breeding strategies. Moreover, precise identification of genomic loci could enable genomic selection and marker-assisted breeding in these fruit species. The role of miRNA cohorts in regulating gene expression during fruit development and ripening is increasingly recognized, with recent discoveries suggesting cross-kingdom therapeutic potential. However, the complex regulatory networks mediated by miRNAs, coupled with the lack of functionally validated miRNA signatures, poorly annotated miRNAomes, and species-specific variation, limit their utility in breeding programmes. Further research to improve miRNA functional annotation and develop comprehensive databases is needed to unlock their potential in fruit improvement. The integration of CRISPR/Cas9 technology represents a powerful tool for trait modification. Despite its promise, several technical challenges remain, including the development of robust transformation protocols, especially in polyploid species with complex genomes, and overcoming issues related to low regeneration rates, epigenetic modifications, and temporal regulation of polygenic traits. Achieving precision in gene editing, improving genomic resources, and addressing regulatory and public perception hurdles will be crucial to realizing the full potential of CRISPR/Cas9 in tropical and subtropical fruit breeding. In addition to technical advancements, the development of web-based resources for tropical and subtropical fruits presents significant opportunities for education, market expansion, and innovation. However, challenges related to logistics, sustainability, and regional diversity must be carefully considered. Single-cell approaches offer unprecedented resolution for trait dissection in tropical and subtropical fruits by uncovering cell-specific expression patterns and rare cell populations. However, their application is constrained by tough tissues, sparse genomic resources, and high technical complexity. Meanwhile, the integration of multi-omics spanning from genomics to metabolomics accelerates gene-to-trait mapping, supported by emerging specialized databases. Yet, challenges persist, including data heterogeneity, limited crop coverage, and the pressing need for standardization. Balancing the benefits and challenges of these resources requires strategic planning, collaboration with multidisciplinary experts, and adaptability to changing environmental and market conditions. Continued investment in these areas will be essential for enhancing the sustainability and resilience of tropical and subtropical fruit crops in the face of global environmental challenges.

## Data Availability

No data were used for the research described in the article and its Supplementary tables.
